# Prolyl oligopeptidase inhibition ameliorates experimental pulmonary fibrosis both in vivo and in vitro

**DOI:** 10.1186/s12931-023-02519-x

**Published:** 2023-08-25

**Authors:** Laura Cucinotta, Deborah Mannino, Giovanna Casili, Alberto Repici, Lelio Crupi, Irene Paterniti, Emanuela Esposito, Michela Campolo

**Affiliations:** Department of Chemical, Biological, Pharmaceutical and Environmental Sciences, University of Messina, 7 Viale Ferdinando Stagno D’Alcontres, 31-98166 Messina, Italy

**Keywords:** Idiopathic pulmonary fibrosis, Prolyl oligopeptidase, Bleomycin, Angiogenesis, Inflammatory

## Abstract

**Background:**

Pulmonary fibrosis is a progressive disease characterized by lung remodeling due to excessive deposition of extracellular matrix. Although the etiology remains unknown, aberrant angiogenesis and inflammation play an important role in the development of this pathology. In this context, recent scientific research has identified new molecules involved in angiogenesis and inflammation, such as the prolyl oligopeptidase (PREP), a proteolytic enzyme belonging to the serine protease family, linked to the pathology of many lung diseases such as pulmonary fibrosis. Therefore, the aim of this study was to investigate the effect of a selective inhibitor of PREP, known as KYP-2047, in an in vitro and in an in vivo model of pulmonary fibrosis.

**Methods:**

The in vitro model was performed using human alveolar A549 cells. Cells were exposed to lipopolysaccharide (LPS) 10 μg/ml and then, cells were treated with KYP-2047 at the concentrations of 1 μM, 10 μM and 50 μM. Cell viability was evaluated by 3-(4,5-dimethylthiazol-2-yl)-2,5-diphenyltetrazolium (MTT) bromide colorimetric assay, while inflammatory protein expression was assessed by western blots analysis. The in vivo model was induced in mice by intra-tracheal administration of bleomycin (1 mg/kg) and then treated intraperitoneally with KYP-2047 at doses of 1, 2.5 and 5 mg/kg once daily for 12 days and then mice were sacrificed, and lung tissues were collected for analyses.

**Results:**

The in vitro results demonstrated that KYP-2047 preserved cell viability, reduced inflammatory process by decreasing IL-18 and TNF-α, and modulated lipid peroxidation as well as nitrosative stress. The in vivo pulmonary fibrosis has demonstrated that KYP-2047 was able to restore histological alterations reducing lung injury. Our data demonstrated that KYP-2047 significantly reduced angiogenesis process and the fibrotic damage modulating the expression of fibrotic markers. Furthermore, KYP-2047 treatment modulated the IκBα/NF-κB pathway and reduced the expression of related pro-inflammatory enzymes and cytokines. Moreover, KYP-2047 was able to modulate the JAK2/STAT3 pathway, highly involved in pulmonary fibrosis.

**Conclusion:**

In conclusion, this study demonstrated the involvement of PREP in the pathogenesis of pulmonary fibrosis and that its inhibition by KYP-2047 has a protective role in lung injury induced by BLM, suggesting PREP as a potential target therapy for pulmonary fibrosis. These results speculate the potential protective mechanism of KYP-2047 through the modulation of JAK2/STAT3 and NF-κB pathways.

**Graphical abstract:**

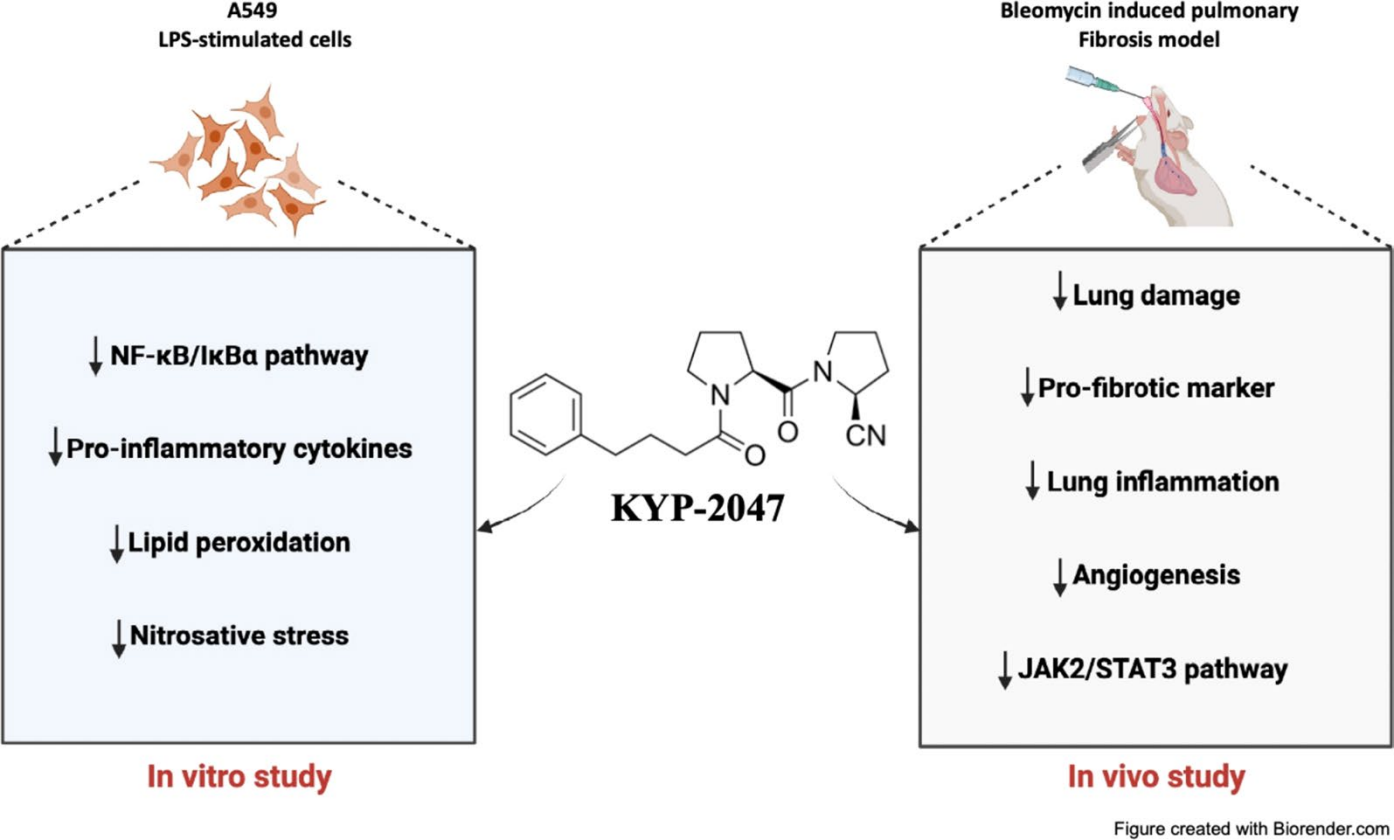

## Introduction

Among all idiopathic interstitial pneumonias, idiopathic pulmonary fibrosis (IPF) is the most common fibrotic lung disease. IPF is a chronic parenchymal lung disease of unknown etiology characterized by a poor prognosis and limited treatment options. 90% of patients experience dyspnea, leading to an extremely low 5-year survival rate of 50% [1, 2].

Although the etiopathogenesis of this disease is unclear, the underlying fibrotic process of this disease is assumed to originate from dysregulation of multiple pathways, including oxidative stress, chemotaxis, inflammation, tissue remodeling, and wound healing in the lung parenchyma [3]. In the early stages of lung injury, neutrophil recruitment to damaged sites occurs, which triggers an immune response and the release of pro-inflammatory cytokines [4, 5]. Moreover, recent evidence demonstrated the role of angiogenesis in the pathogenesis of IPF where the inhibition of vascular remodeling has been shown to attenuate pulmonary fibrosis in animal models [6]. All of these factors contribute to collagen deposition, leading to progressive fibrosis and subsequent loss of lung function [7, 8].

In this context, recent scientific research has identified new molecules strongly involved in angiogenesis and inflammation. Among these, an important role is played by prolyl endo or oligopeptidase (PREP or POP), a proteolytic enzyme belonging to the serine protease family involved in the release of pro-angiogenic and pro-inflammatory molecules [9]. PREP is expressed in different body tissues and in specific cell layers and is implicated in the hydrolysis of proline-containing bioactive peptides, such as angiotensins, arginine-vasopressin, substance P and neurotensin [10]. It is a large intracellular enzyme (molecular mass 80 kDa) that preferentially hydrolyzes proline-containing peptides at the carboxyl end of proline residues involved in the maturation and degradation of peptide hormones and neuropeptides.

In addition to its physiological role, PREP can cleave proline-containing short peptides (less than 3 kDa) that are involved in the activation of the inflammatory response, pathological angiogenesis, and the development of neurodegenerative diseases [9]. Inflammatory stimulation of airway epithelial cells induces the release of PREP-containing exosomes which, starting from collagen fragments, generate proline-glycine-proline (PGP), a chemoattractant of neutrophils [11, 12]. Furthermore, since PREP itself is also present in neutrophils, it plays a role in supporting neutrophilic inflammation, which links PREP to the pathology of many lung diseases such as IPF [13]. Some studies confirm that treatment with PREP inhibitors is beneficial for several disorders [14–17].

Considering the involvement of PREP in inflammatory and pro-angiogenic processes, PREP inhibitors may represent a new therapeutic approach. Specifically, KYP-2047 (4-phenylbutanoyl-l-prolyl-2(S)-cyanopyrolidine) is the most selective and potent inhibitor, having a good ability to reach PREP intracellularly.

Based on this evidence, the aim of this study was to evaluate the beneficial effects of PREP inhibition by KYP-2047 in an in vitro and in vivo model of pulmonary fibrosis.

The in vitro results demonstrated that KYP-2047 preserved cell viability, reduced inflammatory process and modulated lipid peroxidation as well as nitrosative stress. The in vivo results demonstrated that KYP-2047 was able to restore histological alterations reducing lung injury and reduce angiogenesis process and IκBα/NF-κB pathways activation. Moreover, KYP-2047 was able to modulate the JAK2/STAT3 pathway, highly involved in pulmonary fibrosis.

This study demonstrated the involvement of PREP in the pathogenesis of pulmonary fibrosis and that its inhibition by KYP-2047 has a protective role in lung injury, suggesting PREP as a potential target therapy for pulmonary fibrosis.

## Methods

### Materials

KYP-2047 (Sigma, CAS No.: SML020), Lipopolysaccharides (LPS) from Escherichia coli O111:B4 (Sigma, CAS No.: L3012) and all other compounds were purchased by Sigma-Aldrich (Milan, Italy). Each stock solution was prepared in non-pyrogenic saline (0.9% NaCl, Baxter, Milan, Italy).

### In vitro study

#### Cell culture

The human lung alveolar epithelial cell line A-549 (lung adenocarcinoma, ATCC CCL-185) was obtained from the American Type Culture Collection (Manassas, VA, USA). A-549 cells are considered to be alveolar epithelial cells with properties of type II cells, as they were isolated from an alveolar cell carcinoma. A-549 cell line were cultured in 75-cm^2^ flasks in Dulbecco's modified Eagle's medium (DMEM) high glucose supplemented with 10% fetal bovine serum (FBS), 1% antibiotics (100 units/ml penicillin and 100 μg/ml streptomycin) and 1% l-glutamine. Cells were maintained at 37 °C in a humid atmosphere incubator containing 5% of CO_2._

#### Experimental design

Preliminary experiments were performed to evaluate the effects of KYP-2047 on cell viability. For cell viability, 4 × 10^4^ A-549 cells were plated in a volume of 150 µL in 96-well plates (Corning Cell Culture, Tewksbury, MA, USA). After 24 h, the cells were treated with increasing concentrations of KYP-2047 (0.01 μM 0.1 μM, 1 μM, 10 μM and 50 μM) to determine concentrations with minimal cytotoxicity using 3-(4,5-dimethylthiazol-2-yl)-2,5-diphenyltetrazolium bromide (MTT) colorimetric assay. The KYP-2047 concentrations chosen were 1, 10 and 50 μM. In another set of experiments, A-549 cells were seeded on 96-well plates (4 × 10^4^/well) for MTT assay or on 60 mm plates (2 × 10^6^/well) for biochemical analysis. After 24 h cells were exposed to 10 μg/ml of LPS, a membrane component of Gram-negative bacteria, causing a strong pulmonary inflammatory response and oxidative stress [18]. For concentration–response studies, A-549 cells were incubated with 10 μg/ml of LPS and the chosen concentrations of KYP-2047 (1, 10 and 50 μM) or vehicle (basal medium). LPS and KYP-2047 were added simultaneously to the cell culture. For control cells, the medium was replaced by fresh basal medium. After 24 h, supernatants were analyzed for NOx assay (Griess assay) and enzyme-linked immunosorbent assay (ELISA). Then cells were washed with phosphate-buffered saline (PBS), scraped and then pelleted for western blot analysis.

A-549 cells cultures were divided into five groups:

- Control group (Ctr): A-549 cells were cultured with basal medium.
- LPS 10 μg/ml group: A-549 cells were treated with LPS 10 μg/ml for 24 h.
- LPS 10 μg/ml + KYP-2047 1 μM group: A-549 cells were treated simultaneously with LPS 10 μg/ml and KYP-2047 1 μM for 24 h.
- LPS 10 μg/ml + KYP-2047 10 μM group: A-549 cells were treated simultaneously with LPS 10 μg/ml and KYP-2047 10 μM for 24 h.
- LPS 10 μg/ml + KYP-2047 50 μM group: A-549 cells were treated simultaneously with LPS 10 μg/ml and KYP-2047 50 μM for 24 h.

#### Cell viability assay (MTT assay)

Cell viability of A-549 cells was assessed using a mitochondria-dependent live cell dye (tetrazolium dye; MTT) formazan, as previously described [19]. The cultures are pretreated with increasing concentrations of the test compound and incubated with MTT (0.2 mg/ml) for 1 h. Then medium was removed, and cells were suspended in 100 μL dimethylsulphoxide (DMSO) to dissolve the formazan products formed in the cells during the MTT assay and absorbance was measured at 550 nm using a microplate reader.

#### Western blot analysis

Western blot analysis was performed on A-549 cell lysates as previously described [20]. A-549 cells were washed twice with cold PBS, collected, and resuspended in lysis buffer 20 mM Tris–HCl pH 7.5, 10 mM NaF, 150 μL NaCl, 1% Nonidet P-40 and protease inhibitor cocktail (Roche, Monza, Italy). Then, the cells were centrifuged (4 °C for 15 min, 16,000 rpm) and the protein fraction (supernatant) was collected and determined by the Bio-Rad protein assay using bovine serum albumin as a standard. The extracted proteins were denatured by heating at 95 °C for 5 min and equal amounts of proteins were separated on a 12% SDS-PAGE gel and transferred onto a PVDF membrane (Immobilon-P). Finally, the membranes were incubated overnight at 4 °C with the following primary antibodies: anti-inducible nitric oxide synthase (iNOS) (1:500; BD Biosciences #610432), anti-cyclooxygenase-2 (COX-2) (1:500; Santa Cruz Biotechnology sc-376861, Dallas, TX, USA), anti-inhibitor kappa B-alpha (IκB-α) (1:500; Santa Cruz Biotechnology sc-1643, Dallas, TX, USA), anti-nuclear factor kappa-light-chain-enhancer of activated B cells (NF-κB) (1:500; Santa Cruz Biotechnology sc-8008, Dallas, TX, USA) and anti-interleukin-18 (IL-18) (1:500; Santa Cruz Biotechnology sc-7954, Dallas, TX, USA). To ensure that equal amounts of protein lysate were loaded, the membranes were incubated with β-actin antibody (1:500; sc-47778). Signals were detected with Advanced Chemiluminescence Detection System (ECL) reagent according to the manufacturer's instructions (Thermo Fisher, Waltham, MA, USA). The relative expression of the protein bands was quantified by densitometry with the BIORAD ChemiDocTMXRS + software.

#### Quantitative real-time polymerase chain reaction (Q-RT-PCR)

Quantitative Real time PCR was performed as previously described [21]. Total RNA was extracted from cultivated cells using Trizol reagent (Thermo Fisher, USA). First-strand cDNAs were synthesized using the PrimeScript™ RT reagent Kit with gDNA Eraser (Perfect Real Time) (Takara, Shiga, Japan). Quantitative RT-PCR was performed with the StepOnePlus Real-Time PCR System (Thermo Fisher Scientific). All samples were run in duplicate, and the results were averaged and normalized to the expression level of GAPDH. The CT value (amplification power curve inflection point) was obtained, ∆Ct = CT (target gene) − CT (internal reference), ∆∆Ct = ∆Ct (treatment group) − ∆Ct (control group); the relative expression of target genes was calculated using 2 − ∆∆Ct. This analysis was performed to measure E-cadherin and N-cadherin mRNA expressions. PCR primers for all analyzed genes were:

- E-cadherin (forward: GCCCCGCCTTATGATTCTCTGC; reverse: CTCGCCGCCTCCGTACATGTC).
- N-cadherin (forward: 5′ TTTGATGGAGGTCTCCTAACACC3′; reverse: 5′ ACGTTTAACACGTTGGAAATGTG3′)
- GAPDH (forward: ACCCATCACCATCTTCCAGGAG; reverse: GAAGGGGCGGAGATGATGAC).

#### Elisa assay

ELISA kit was used to detect the levels of PREP (BT-LAB bioassay technology laboratory Catalog.: E2664Mo) and pro-inflammatory cytokine production tumor necrosis factor-α (TNF-α) (TNF-α ELISA Kit Abcam ab100654) in cell culture supernatant according to the manufacturer’s protocols. Briefly, the samples (50 µL) were added in anti-TNF-α and anti-PREP biotin-coated well plates and incubated at 37 °C for 60 min; plates were washed five times with washing buffer 1X solution. Subsequently, 100 µL of the horseradish peroxidase (HRP) was added to each well and incubated for 30 min at room temperature. Plate was washed five times with washing buffer 1X and TMB (3,3′5,5′ tetramethyl-benzidine) substrate solution (100 µL) was added and incubated for 15 min at room temperature. Finally, 100 µL of stop solution was added and the concentrations of PREP and TNF-α were determined spectrophotometrically at an absorbance of 450 nm and interpolated with a standard curve.

#### NOX Assay

The effect of KYP-2047 on NO production by A-549 LPS-stimulated cells was investigated by colorimetric Griess reaction [22]. Cell culture supernatant from each group was collected from plates and total nitrite levels, as an indicator of nitric oxide (NO) synthesis, were measured. Briefly 100 µL of supernatant was transferred to microplate wells and 100 µL Griess reagent (1% sulfanilamide and 0.1% *N*-naphthylethyl-ethylenediamine dihydrochloride in 5% phosphoric acid were mixed at a ratio of 1:1) was added, respectively. The mixture was incubated for 10 min at room temperature. The absorbance was measured by a microplate reader at 540 nm, and nitrite concentration was determined using a curve calibrated on sodium nitrite standards.

### In vivo study

#### Animals

Male adult CD1 (25–30 g; 6–8 weeks of age) were purchased from Envigo (Milan, Italy). Animals were placed in a controlled environment at consistent temperature (22 °C ± 2 °C). Animals were fed with a standard diet and water ad libitum under pathogen-free conditions with a 12 h light/12 h dark cycle. After 7 days of adaptation, animal model was established. The animal study was approved by the University of Messina Review Board following Italian regulations on the use of animals (D.M.116192) and Directive legislation (EU) (2010/63/EU) amended by Regulation (EU) 2019/1010.

#### Bleomycin (BLM)—induced pulmonary fibrosis model

The induction of lung injury by bleomycin (BLM) was performed as previously described [23]. Mice were anesthetized and then, BLM sulfate (1 mg/kg body weight) was administered by a single intratracheal injection. A volume of 100 μL was injected at the end of expiration to ensure delivery to the distal airways. This was immediately followed by 300 μL of air. One hour after surgery, KYP-2047 (1 mg/kg, 2.5 mg/kg and 5 mg/kg) was administered intraperitoneally. The treatment was repeated daily for 12 days. Mice in the control groups received intratracheal injection of normal saline. At the end of the experiment, on day 12, the mice were sacrificed by euthanasia using isoflurane inhalation and the middle lobe of the right lung was collected by surgical procedure and processed for histological, immunohistochemical and western blot analysis.

This experimental mouse model of BLM-induced pulmonary fibrosis is widely used in the evaluation of potential antifibrotic agents. Several studies highlighted that single bleomycin instillation effectively replicates some of the specific pathogenic molecular changes associated with IPF. Indeed, overproduction of reactive species and activation of fibroblasts caused by BLM administration are typical features of the human disease [24–26].

#### Experimental groups

Mice were randomized into the following experimental groups (n = 10):

- Sham + vehicle group. Mice received intratracheal administration of saline and after 1 h were treated with vehicle (saline) intraperitoneally daily for 12 days.
- BLM + vehicle group. Mice received BLM (1 mg/kg) administration and were treated intraperitoneally daily with the vehicle (saline) for 12 days.
- BLM + KYP-2047 1 mg/kg. Mice received BLM administration and treated with KYP-2047 1 mg/kg, immediately 1 h after BLM instillation every day starting from day 1 until day 12.
- BLM + KYP-2047 2,5 mg/kg. Mice received BLM administration and treated with KYP-2047 2,5 mg/kg, immediately 1 h after BLM instillation every day starting from day 1 until day 12.
- BLM + KYP-2047 5 mg/kg. Mice received BLM administration and treated with KYP-2047 5 mg/kg, immediately 1 h after BLM instillation every day starting from day 1 until day 12.

KYP-2047 was dissolved in saline (0.001% DMSO) and administered according to the bibliography [27]. The doses of KYP-2047 (1 mg/kg, 2.5 mg/kg and 5 mg/kg) used for the experiment were based on previous in vivo study made in our laboratories [13, 28]. The BLM + KYP-2047 1 mg/kg was only subjected to histological evaluation, Masson’s trichrome and Sirius red stains, because it did not induce any beneficial effect; therefore, we decided to continue by analyzing KYP-2047 only at the doses of 2.5 and 5 mg/kg.

#### Histological analysis

To assess the severity of lung injury, lung samples were analyzed as previously described [13]. Briefly, lung samples from each experimental group were fixed in buffered formalin with 10% (w/v) PBS for 24 h, followed by dehydration, paraffin embedding, and cut into 7 μM thick sections. Sections were placed on a polylysine-coated slide, immersion in xylene for deparaffinization, rehydration via alcohol gradient, and finally staining with hematoxylin and eosin (H/E). To measure the extent of pulmonary fibrosis, each field was individually assessed for the degree of lung damage and classified on a scale of 0 to 8 as follows: grade 0, normal lung; grade 1, minimal fibrous thickening of the alveolar or bronchiolar walls; grade 3, moderate thickening of the alveolar or bronchiolar walls with no obvious damage to the pulmonary architecture; grade 5, formation of small fibrous masses with definitive damage to the lung structure; grade 7, large fibrotic areas and severely altered lung structure; grade 8, total fibrous obliteration of the fields. Grades 2, 4 and 6 were used as intermediates of the above criteria [29]. All sections were evaluated by a single blinded investigator. Images were shown at 20× magnification (50 μM of the Bar scale) using a Nikon Eclipse Ci-L microscope.

#### Proteins Concentration and cell counts in bronchoalveolar lavage fluid (BALF)

The cell count in BALF was carried out as previously described [30]. Briefly, BALF was collected by cannulating the trachea and lavaging the lung twice with 0.7 mL of phosphate- buffered saline (PBS). The washing solution were removed by aspiration and BALF was centrifugated at 800 rpm. The supernatant was stored at − 20 °C, while the pelleted cells were resuspended in PBS. Then, the total cells in BALF were enumerated by counting with a hemocytometer in the presence of the trypan blue stain. For differential cell counting, Wright’s Giemsa stain was performed, and the leukocyte and macrophage populations present in BALF were counted. After staining, the differential count was carried out by the standard morphological protocol under a light microscope. To measure the pro-inflammatory cytokines, the levels of IL-6 and IL-1β and were detected using ELISA, according to the manufacturer’s protocols (Abcam cat.: ab100713 for IL-6; Invitrogen, cat.:BMS6002 for IL-1β).

#### Masson’s trichrome and Sirius red stains

To further evaluate the degree of fibrosis collagen accumulation in the lung tissues, sections were stained with Masson trichrome and Sirius red according to the manufacturer’s protocols (Bio-Optica, Milan, Italy), as previously described [31]. Images were shown at 20× magnification (50 μM of the Bar scale) using a Nikon Eclipse Ci-L microscope.

#### Western blot analysis

Lung tissues were homogenized, and western blots were performed as already mentioned [32]. After protein extraction from lung tissues, lysates were used for the detection of iNOS, COX-2, IκBα, TNF-α, Janus kinase 2 (p-JAK2), signal transducer and activator of transcription 3 (p-STAT3), α-smooth muscle actin (α-SMA), endothelial nitric oxide synthase (eNOS) and cluster of differentiation 34 (CD34) at the cytosolic level and the detection of NF-κB at the nuclear level. Membranes were incubated at 4 °C overnight with each of the following primary antibodies: anti-iNOS (1:500; BD Biosciences #610432), anti-COX-2 (1:500; Santa Cruz Biotechnology sc-376861, Dallas, TX, USA), anti-IκB-α (1:500; Santa Cruz Biotechnology sc-1643, Dallas, TX, USA), anti-TNF-α (1:500; Santa Cruz Biotechnology sc-52746, Dallas, TX, USA), anti-NF-κB (1:500; Santa Cruz Biotechnology sc-8008, Dallas, TX, USA),, anti-α-SMA (1:500; Santa Cruz Biotechnology, sc-53015, Dallas, TX, USA), anti-p-JAK2 (1:500; Santa Cruz Biotechnology sc-16556, Dallas, TX, USA), anti-p-STAT3 (1:500; Cell Signaling #8768P, Danvers, MA, USA), anti-eNOS (1:500; Santa Cruz Biotechnology sc-654, Dallas, TX, USA) and anti-CD34 (1:500; Santa Cruz Biotechnology sc-74499, Dallas, TX, USA). Then, membranes were incubated with peroxidase-conjugated bovine secondary antibody (Jackson ImmunoResearch, West Grove, PA, USA; 1:2000) for 1 h at room temperature. Signals were detected with an enhanced chemiluminescence detection system (Super-SignalWest Pico Chemiluminescent Substrate, Pierce, Monza, Italy). The relative expression of the protein bands was quantified by densitometry with Bio-Rad ChemiDoc XRS software (Bio-Rad, Milan, Italy) and standardized to β-actin or lamin A/C levels.

#### Immunohistochemical analysis

Transforming growth factor β (TGF-β), an important profibrotic growth factor and vascular endothelial growth factor (VEGF), a key angiogenic mediator of pulmonary fibrosis were determined by immunohistochemistry in lung tissues as previously described [33]. Lung sections were incubated overnight with anti-TGF-β (Santa Cruz Biotechnology; 1:100 sc-130348 in PBS, v/v, MA, USA) and anti-VEGF polyclonal antibody (Santa Cruz Biotechnology; 1:100 sc-7269 in PBS, v/v, MA, USA). At the end of the incubation with the primary antibodies, the sections were washed with PBS and incubated with a secondary antibody (Santa Cruz Biotechnology, Dallas, TX, USA) for 1 h at room temperature. The reaction was revealed by a chromogenic substrate (brown DAB), and counterstaining with nuclear fast-red. Images were collected using a microscope and AxioVision software. For graphical display of densitometric analyses, % positive staining (brown staining) was measured by computer-assisted color image analysis (Nikon Eclipse Ci-L microscope). The percentage area of immunoreactivity (determined by the number of positive pixels) was expressed as a percentage (%) of the total tissue area (red staining) at 20× magnification.

#### Quantitative real-time polymerase chain reaction (Q-RT-PCR)

Quantitative real time PCR was performed as previously described [21]. Total RNA was extracted from lung tissues using Trizol reagent (Thermo Fisher, USA). First-strand cDNAs were synthesized using the PrimeScript™ RT reagent Kit with gDNA Eraser (Perfect Real Time) (Takara, Shiga, Japan). Quantitative RT-PCR was performed with the StepOnePlus Real-Time PCR System (Thermo Fisher Scientific). All samples were run in duplicate and the results were averaged and normalized to the expression level of GAPDH. The CT value (amplification power curve inflection point) was obtained, ∆Ct = CT (target gene) − CT (internal reference), ∆∆Ct = ∆Ct (treatment group) − ∆Ct (control group); the relative expression of target genes was calculated using 2 − ∆∆Ct. This analysis was performed to measure type I collagen, TGF-β1, VEGF, E-cadherin and N-cadherin mRNA expressions in the lung samples. PCR primers for all analyzed genes were:

- Type I collagen (forward 5′-ACGACAGAAGGAGAGCAGAAG-3’; reverse 5′-ATGTCCACCAGGGTCTCAATC-3′)
- TGF-β1 (forward: CAGCAACAATTCCTGGCGATA; reverse: GCTAAGGCGAAAGCCCTCAAT)
- VEGF (forward 5′-GAGCAGAAGTCCCATGAAGTGA-3′ and reverse 5′-CACAGGACGGCTTGAAGATGT-3′).
- E-cadherin (forward: GCCCCGCCTTATGATTCTCTGC; reverse: CTCGCCGCCTCCGTACATGTC).
- N-cadherin (forward: 5′ TTTGATGGAGGTCTCCTAACACC3′; reverse: 5′ ACGTTTAACACGTTGGAAATGTG3′)
- GAPDH (forward: ACCCATCACCATCTTCCAGGAG; reverse: GAAGGGGCGGAGATGATGAC).

### Statistical analysis

All values are expressed as the mean ± standard error of the mean (SEM) of *n* observations. Each analysis was performed three times, with three samples replicated for each one. The results were analyzed with GraphPad 9 software by one-way analysis of variance (ANOVA), followed by a Bonferroni post hoc test for multiple comparisons. A *p*-value of less than 0.05 was considered significant.

## Results

### In vitro results

#### Effect of KYP-2047 on cell viability

**Fig. 1:**
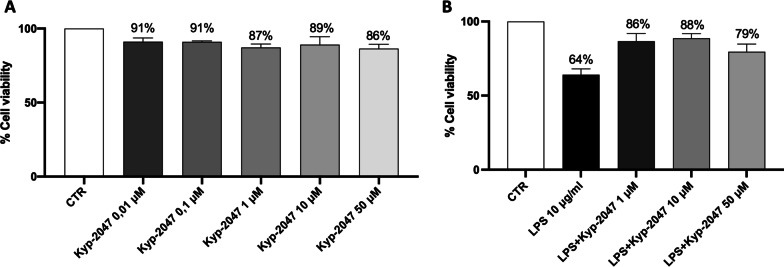
Effect of KYP-2047 on A-549 cell viability. MTT assay revealed that KYP-2047 at the concentrations of 0.01 μM, 0.1 μM, 1 μM, 10 μM and 50 μM did not exert any cytotoxic effects on A549 cells (**A**). The panel **B** showed that LPS group decreased cell viability, while the treatment with KYP-2047 at 1 μM, 10 μM and 50 μM preserved cell viability (**B**). Data are representative of at least three independent experiments

An MTT assay was performed to evaluate the concentration with minimal toxicity of KYP-2047 on the viability A549 cells. As shown in Fig. 1, KYP-2047 at the concentrations of 0.01, 0.1, 1, 10, 30 and 50 μM did not exert any cytotoxic effects on A549 cells. Thus, the effects of KYP-2047 on A549 cells were not attributable to cytotoxic effects (Fig. 1A). Then, we evaluated the effect of KYP-2047 on A549 cells viability following LPS-stimulation. Our results demonstrated that the LPS group was characterized by a decrease in cell viability compared to the control group, while treatment with KYP-2047 at 1, 10 and 50 μM preserved cell viability (Fig. 1B).

#### The levels of PREP in A549-LPS stimulated cells

**Fig. 2:**
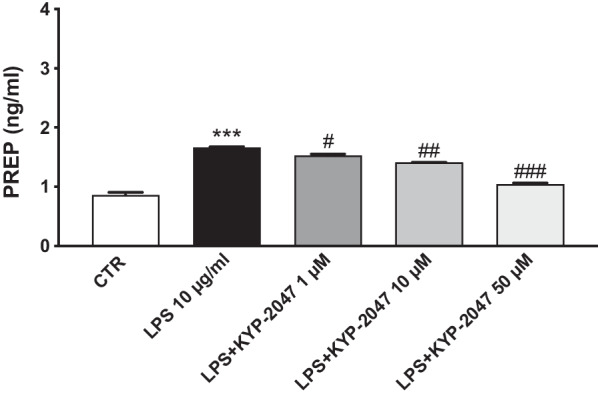
Effect of KYP-2047 on PREP in A-549 LPS-stimulated cells. ELISA assay revealed an increase of PREP levels in LPS-stimulated cells compared to control cells. KYP-2047 at the concentrations of 0. 1 μM, 10 μM and 50 μM significantly reduced PREP levels compared to LPS-stimulated cells. Data are representative of at least three independent experiments. One way ANOVA test ***p < 0.001 vs CTR; ^#^p < 0.05 vs LPS; ^##^p < 0.01 vs LPS; ^###^p < 0.001 vs LPS

To clearly demonstrate the role of PREP in inflammatory process we performed ELISA assay in LPS-stimulated A549 cells. Our results demonstrated basal levels of PREP in control cells, while a significant increase in PREP levels was observed under LPS stimulation. Treatment with KYP-2047 at concentrations of 1 μM, 10 μM and 50 μM significantly reduced PREP levels compared to LPS-stimulated cells (Fig. 2).

#### KYP-2047 modulated NF-κB/IκBα expression and pro-inflammatory cytokines’ expression

**Fig. 3:**
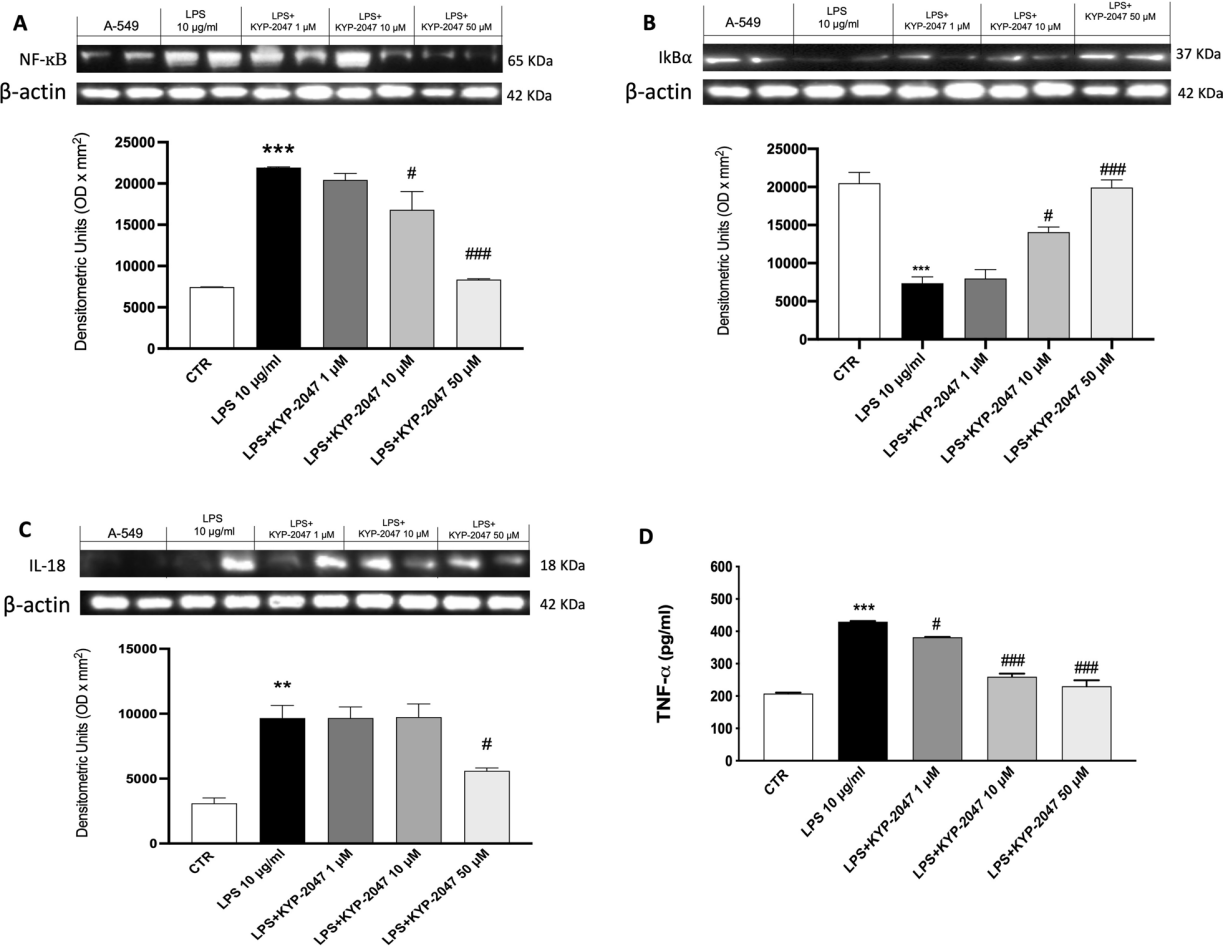
Effect of KYP-2047 on IκBα/NF-κB pathway and inflammation. Western blot analyses showed that KYP-2047 was able to modulate IκBα/NF-κB (respectively **B** and **A**); additionally, treatment with KYP-2047 reduced IL-18 (**C**) compared to LPS group. ELISA kit showed that KYP-2047 reduced TNF-α (**D**) expression compared to LPS group. Data are representative of at least three independent experiments. One way ANOVA test **p < 0.01 vs CTR; ***p < 0.001 vs CTR; ^#^p < 0.05 vs LPS; ^###^p < 0.001 vs LPS

LPS exposure induces an intense inflammatory response [34], therefore, to test the anti-inflammatory effect of KYP-2047, we measured the expression of NF-κB and its inhibitor IκBα proteins. As shown in Fig. 3A, B, LPS significantly induced NF-κB expression and IκBα degradation in A549 cells. KYP-2047 at concentrations of 10 and 50 μM significantly reduced the expression of NF-κB and IκBα compared to the control group. On the contrary, KYP-2047 at concentration of 1 μM did not show any effect. To further investigate the anti-inflammatory effects of KYP-2047, we decided to evaluate the expression of IL-18 by Western blot analysis in cell lysates and TNF-α by ELISA kit in cell culture supernatant. Our results showed a significant increase of IL-18 and TNF-α after LPS-stimulation, compared to the control cells. However, the treatment with KYP-2047 reduced the expression of IL-18 at the concentration of 10 μM compared to the control cells (Fig. 3C). Moreover, KYP-2047 reduced TNF-α expression at all the concentration (Fig. 3D).

#### KYP-2047 modulated lipid peroxidation and nitrosative stress

**Fig. 4:**
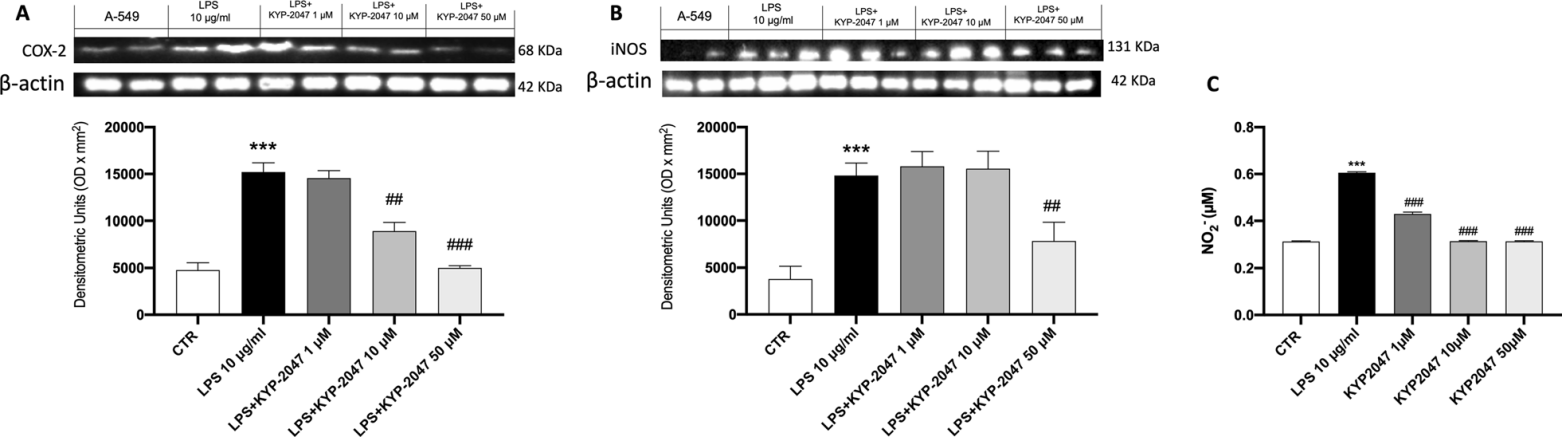
Effect of KYP-2047 on lipid peroxidation and nitrosative stress. Western Blot analysis showed an important modulation of COX-2 (**A**) and iNOS (**B**). Additionally, KYP-2047 treatments modulated nitrosative stress, reducing NO-2 levels (**C**). Data are representative of at least three independent experiments. One way ANOVA test ***p < 0.001 vs CTR; ^##^p < 0.01 vs LPS; ^###^p < 0.001 vs LPS

Exposure to LPS also induces lipid peroxidation which involves the activation of COX-2 and iNOS. Our results showed a marked increase in COX-2 and iNOS expression after LPS stimulation compared to the control cells, However, KYP-2047 treatments reduced both expressions (Fig. 4A and B). Nitric oxide (NO) is mainly produced by iNOS, especially during inflammatory lung states. A549 cells treated with LPS 10 μg/ml showed a higher extracellular NO level than untreated A549 cell culture supernatants. In contrast, NO levels in cells supernatant treated KYP-2047 at concentrations of 1 μM, 10 μM and 50 μM were significantly reduced compared to control cells (Fig. 4C).

#### KYP-2047 reduced epithelial-mesenchymal transition (EMT) in A549 LPS- stimulated cells

**Fig. 5:**
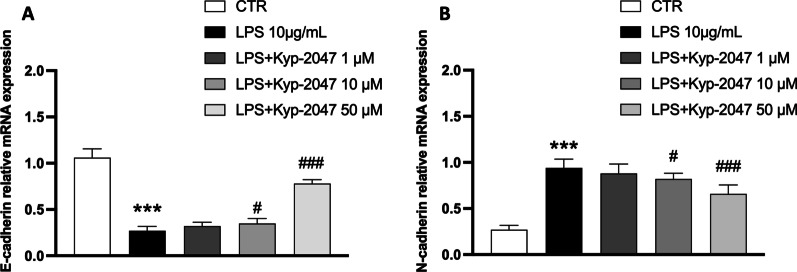
Effect of KYP-2047 on EMT markers. qRT-PCR was used to measure mRNA levels of EMT-related markers such as E-cadherin and N-cadherin after KYP-2047 treatment. Epithelial marker E-cadherin mRNA decreased significantly after LPS stimulation compared to control cells (**A**), while N-cadherin mRNA significantly increased after LPS stimulation (**B**). KYP-2047 treatments restored their gene levels compared to LPS-stimulated cells. Data are representative of at least three independent experiments. One way ANOVA test ***p < 0.001 vs CTR; ^##^p < 0.01 vs LPS; ^###^p < 0.001 vs LPS

As shown in Fig. 5A, B, qRT-PCR analysis demonstrated that among EMT-related markers, E-cadherin gene levels were down-regulated, and N-cadherin gene levels were up-regulated in A549-LPS stimulate. However, EMT was markedly reversed following KYP-2047 treatment. In fact, cells treated with KYP-2047 at concentrations of 10 and 50 significantly restored e-cadherin gene levels (Fig. 5A) and reduced n-cadherin gene levels (Fig. 5B) compared to LPS-stimulated cells.

### In vivo results

#### Histological effects of KYP-2047 on lung damage induced by BLM

**Fig. 6:**
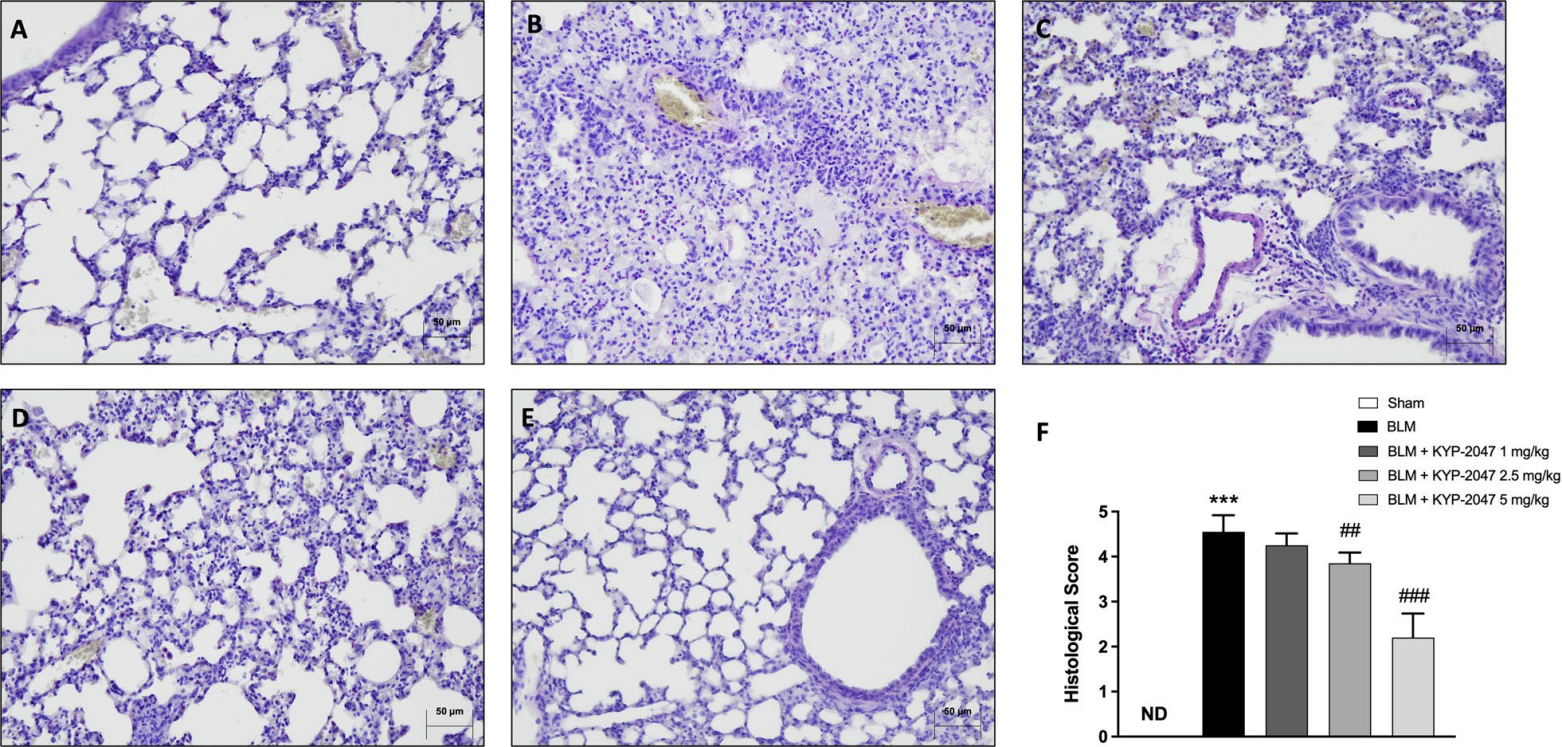
Effect of KYP-2047 on histological damage. H&E staining of Sham group (**A**), and BLM group (**B**), see histological score (**F**). KYP-2047 treatments after BLM: KYP-2047 1 mg/kg (**C**), KYP-2047 2.5 mg/kg (**D**) and KYP-2047 5 mg/kg (**E**); see histological score (**F**). Images were shown at ×20 magnification. Data are representative of at least three independent experiments. One way ANOVA test ***p < 0.001 vs Sham; ^##^p < 0.01 vs BLM; ^###^p < 0.001 vs BLM

H/E staining was performed to evaluate histopathological changes following bleomycin damage in the middle lobe of the right lung. In the sham group we observed intact and clear alveoli, normal interstitium (Fig. 6A, see histological score F). On the contrary, in the BLM-induced group we observed inflammatory and fibrotic changes such as the destruction of lung alveoli and inflammatory cell infiltration in the lung tissues (Fig. 6B, see histological score F). However, KYP-2047-treated mice at doses of 2.5 and 5 mg/kg (Fig. 6D, E, see histological score F) showed a less severe pattern of lung lesions, consisting of moderate inflammation and mild fibrosis compared to BLM mice. KYP-2047 treatment at a dose of 1 mg/kg, did not provide any protection (Fig. 6C, see histological score F).

#### Effect of KYP-2047 on inflammatory cells and pro-inflammatory cytokines in BALF

**Fig. 7:**
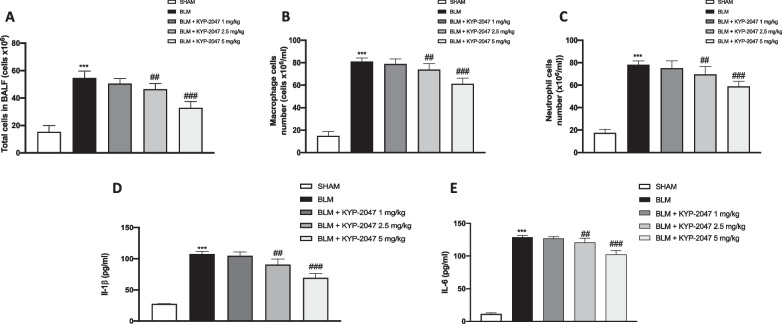
Effect of KYP-2047 on cell infiltration and proinflammatory cytokine expression in BALF. Total cell number (**A**), Macrophages (**B**), Neutrophils (**C**). Levels of proinflammatory cytokine: IL-1β (**D**), IL-6 (**E**). Data are representative of at least three independent experiments. One way ANOVA test ***p < 0.001 vs Sham; ^##^p < 0.01 vs BLM; ^###^p < 0.001 vs BLM

To determine whether KYP-2047 was able to reduce cellular infiltration, we measured inflammatory cell counts in the BALF. We found substantial increases in total cell (Fig. 7A), macrophage (Fig. 7B), and neutrophil (Fig. 7C) counts in BALF taken from BLM-treated animals compared to Sham mice. The number of inflammatory cells in BALF was significantly reduced following treatment with KYP-2047 at doses of 2.5 mg/kg and 5 mg/kg (Fig. 7A–C). Furthermore, in the BALF of animals of each experimental group we examined the levels of the pro-inflammatory cytokines IL-1β and IL-6. The levels of IL-1β (Fig. 7D) and IL-6 (Fig. 7E) were significantly increased in the BLM group compared to Sham mice. In contrast, cytokine release in BALF was markedly reduced in KTP-2047-treated mice at doses of 2.5 mg/kg and 5 mg/kg (Fig. 7D, E).

#### Role of KYP-2047 treatment in collagen content reduction on pulmonary damage induced by BLM

**Fig. 8:**
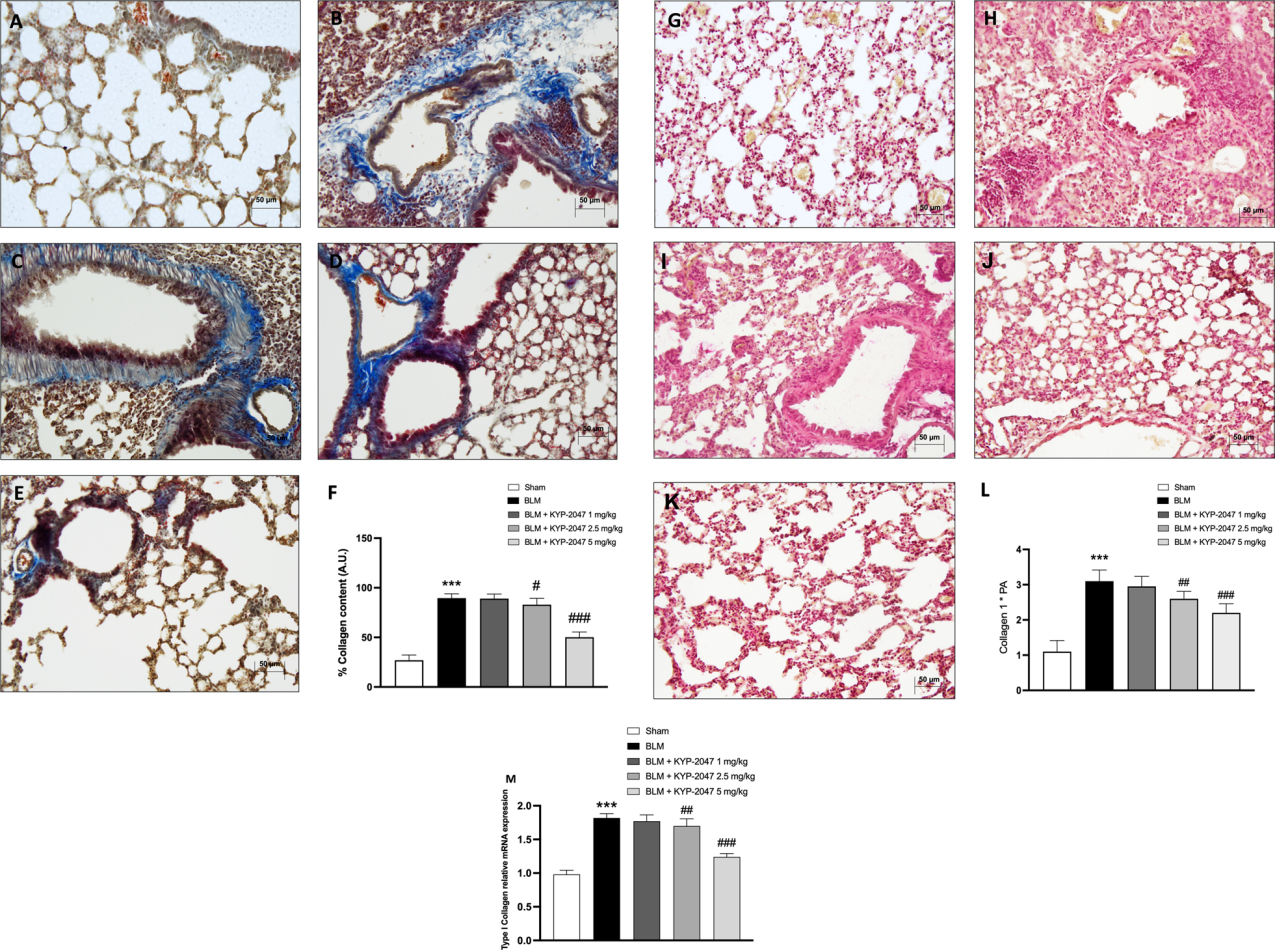
Effect of KYP-2047 on collagen content. Masson’s trichrome staining of Sham group (**A**), and BLM group (**B**); KYP-2047 treatments after BLM: KYP-2047 1 mg/kg (**C**), KYP-2047 2.5 mg/kg (**D**) and KYP-2047 5 mg/kg (**E**). See percentage collagen content (**F**). Sirius Red staining of Sham group (**G**), and BLM group (**H**). KYP-2047 treatments after BLM: KYP-2047 1 mg/kg (**I**), KYP-2047 2.5 mg/kg (**J**) and KYP-2047 5 mg/kg (**K**). See score (**L**). Images were shown at ×20 magnification. Pulmonary type I collagen mRNA was measured by real-time RT-PCR (M). Data are representative of at least three independent experiments. One way ANOVA test ***p < 0.001 vs Sham; ^#^p < 0.05 vs BLM; ^##^p < 0.01 vs BLM; ^###^p < 0.001 vs BLM

A key marker of pulmonary fibrosis is an excessive collagen deposition [35]; therefore, we performed Masson’s trichrome and Sirius Red staining to examine the amount of collagen deposition in the middle lobe of the right lung. Masson’s trichrome staining showed a large amount of blue collagen deposition in the pulmonary interstitium in BLM-induced mice as compared to sham group (respectively, Fig. 8B and A, see percentage of collagen content Fig. 8F). However, the collagen content was significantly reduced after treatments with KYP-2047 (Fig. 8D and E, see percentage of collagen content Fig. 8F) Also in this analysis, treatment at a dose of 1 mg/kg, did not provide any beneficial effect (Fig. 8C, see percentage of collagen content Fig. 8F).

At the same way, Sirius Red staining showed an increase in red collagen fibers in BLM-induced mice as compared to the sham group (respectively, Fig. 8H and G, see percentage of collagen content Fig. 8L). On the contrary, KYP-2047 treatments at doses of 2.5 and 5 mg/kg reduced the collagen content (Fig. 8J and K, see percentage of collagen content Fig. 8L). KYP-2047 treatment at dose of 1 mg/kg did not provide any beneficial effect (Fig. 8I, see percentage of collagen content Fig. 8L). Moreover, as expected qRT-PCR revealed that pulmonary type I collagen mRNA was significantly upregulated in BLM-treated mice, compared to the sham group (Fig. 8M).

**Fig. 9:**
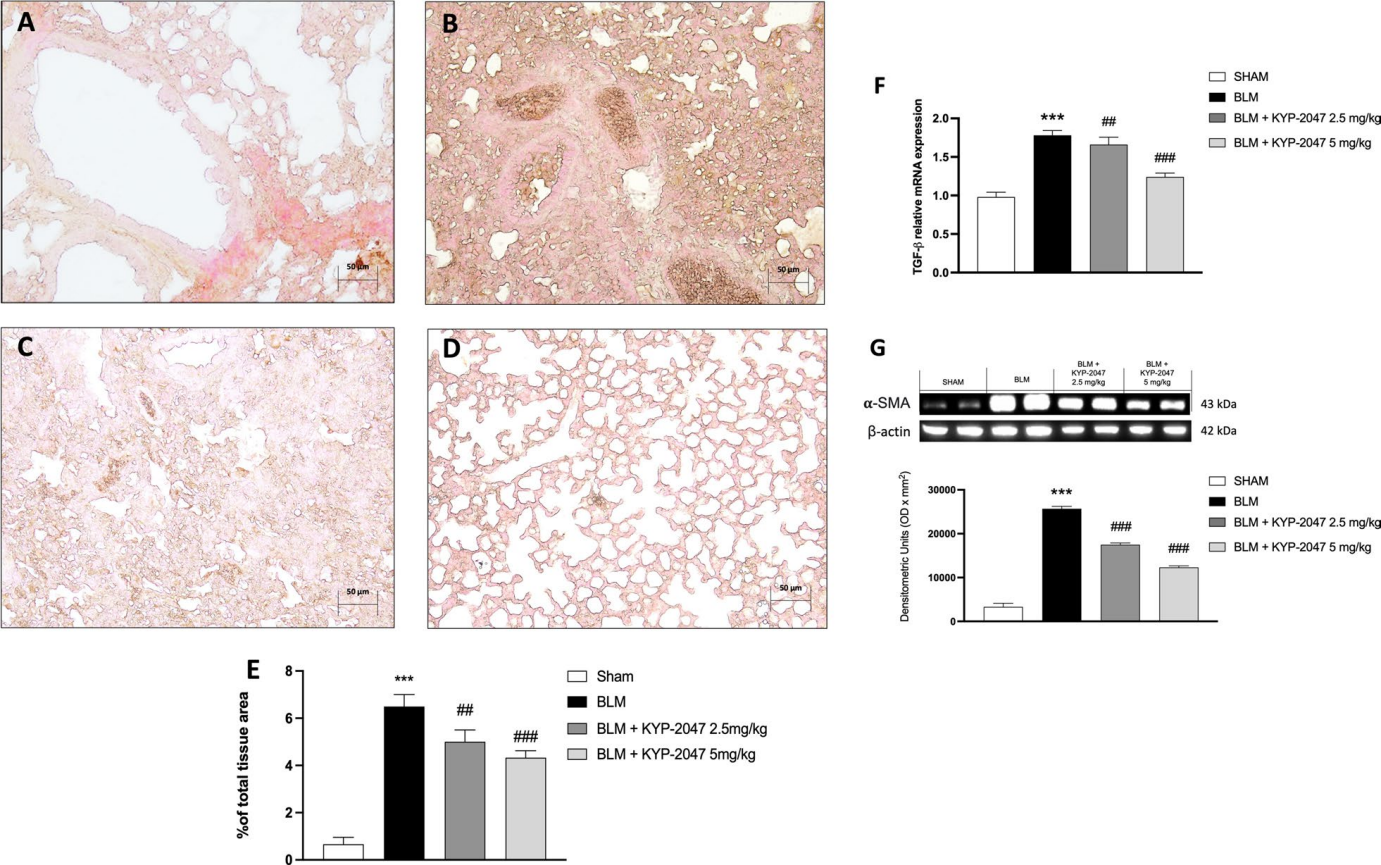
Effect of KYP-2047 on TGF-β and α-SMA markers. Immunohistochemical analyses of TGF-β: Sham group (**A**), and BLM group (**B**); KYP-2047 treatments after BLM: KYP-2047 2.5 mg/kg (**C**) and KYP-2047 5 mg/kg (**D**). See percentage of total tissue area (**E**). Images were shown at ×20 magnification. Pulmonary TGF-β mRNA was measured by qRT-PCR (**F**). Western blot analyses showed an important decrease of α-SMA after KYP-2047 treatments compared to BLM group (**G**). Data are representative of at least three independent experiments. One way ANOVA test ***p < 0.001 vs Sham; ^##^p < 0.01 vs BLM; ^###^p < 0.001 vs BLM

Several studies have shown that TGF-β plays an important role in the pathogenesis of pulmonary fibrosis [36]. In fact, TGF-β is known to be a key mediator of collagen synthesis in the development of pulmonary fibrosis and its expression appears to be upregulated in the lung disease [37]. In this study, through immunohistochemical analysis, the upregulation of TGF-β was confirmed in the middle lobe of the right lung samples of BLM group compared to control group (respectively, Fig. 9B and A, see TGF-β positive score Fig. 9E). Interestingly, lung samples from KYP-2047 treated group, at both doses of 2.5 and 5 mg/kg, significantly reduced TGF-β positive staining (Fig. 9C and D, see TGF-β positive score Fig. 9E). Moreover, as expected the expression of pulmonary TGF-β mRNA was significantly upregulated in BLM-treated mice (Fig. 9F). We also evaluated α-SMA expression as marker of collagen accumulation [38]; our result confirmed a significant increase of α-SMA expression in BLM-induced group compared to the sham group. Again, treatment with KYP-2047 significantly reduced α-SMA expression, confirming the ability of KYP- 2047 to reduce collagen content (Fig. 9G).

#### Role of KYP-2047 on fibrosis marker

**Fig. 10:**
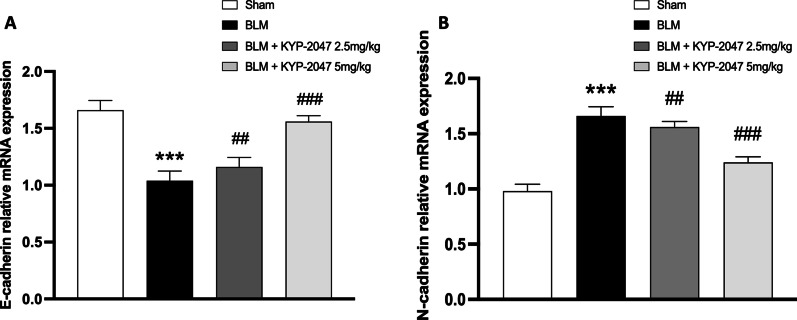
Effect of KYP-2047 on fibrosis markers: E-Cadherin (**A**) and N-Cadherin (**B**). RT-PCR analysis showed a decrease of N-Cadherin and an increase of E-Cadherin gene levels in BLM group compared to the sham group. KYP-2047 treatments restored their gene levels. Data are representative of at least three independent experiments. One way ANOVA test ***p < 0.001 vs Sham; ^##^p < 0.01 vs BLM; ^###^p < 0.001 vs BLM

The EMT of fibroblasts to myofibroblasts is one of the most important mechanisms of fibrosis and plays a pivotal role in the development of IPF [39]. For these reasons, we evaluated some of common indicators of EMT, such as N-cadherin and E-cadherin. qRT-PCR analysis demonstrated a significant decrease of E-cadherin gene levels (Fig. 10A) and a significant increase of N-cadherin gene levels (Fig. 10B) in BLM mice. The treatment with KYP-2047 at doses of 2.5 mg/kg and 5 mg/kg restored EMT marker gene levels.

#### Role of KYP-2047 on modulation of angiogenesis in lung

**Fig. 11:**
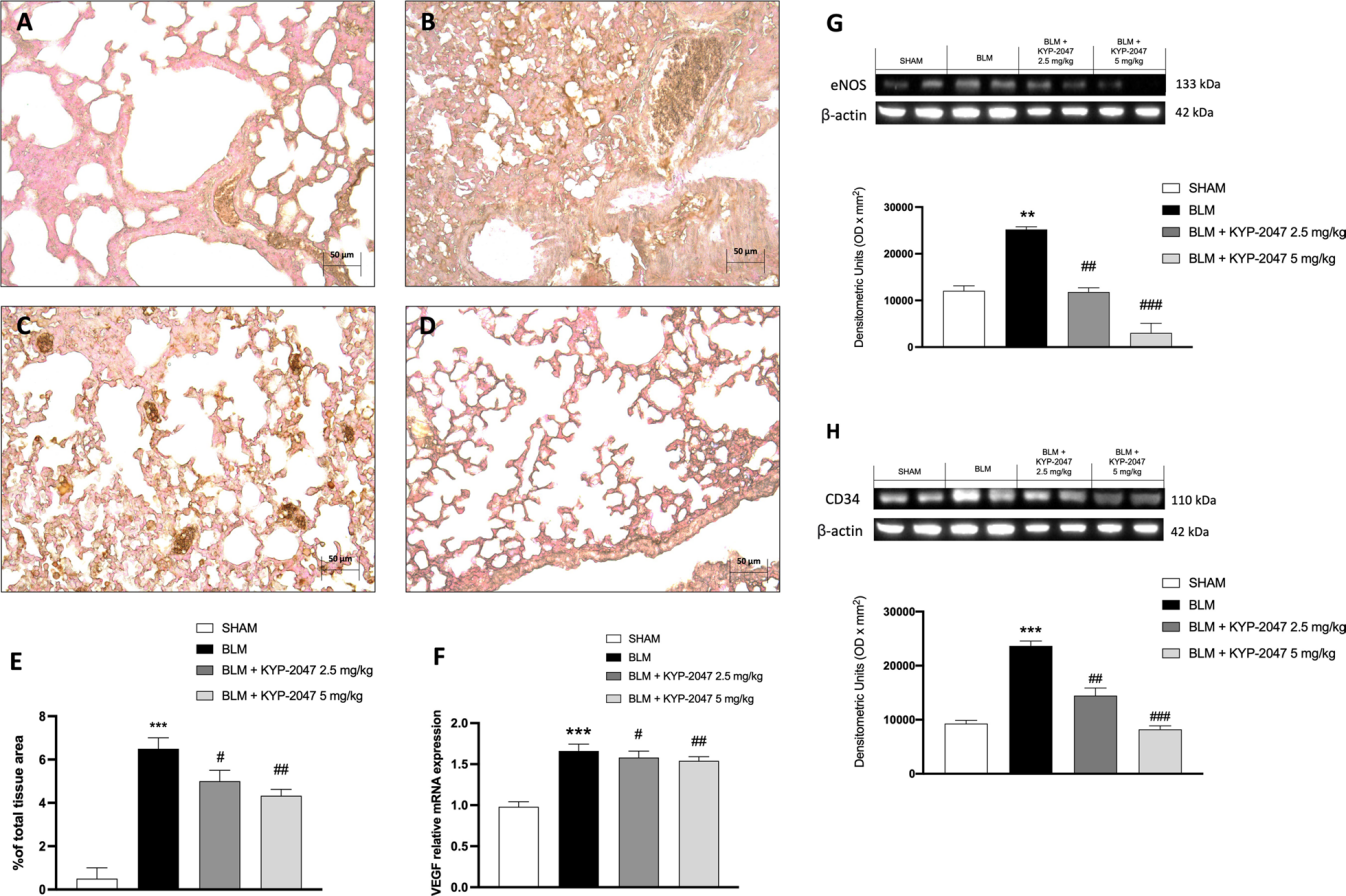
Effect of KYP-2047 on angiogenesis. Immunohistochemical analyses of VEGF: Sham group (**A**), and BLM group (**B**). KYP-2047 treatments after BLM: KYP-2047 2.5 mg/kg (**C**) and KYP-2047 5 mg/kg (**D**). See percentage of total tissue area (**E**). Images were shown at ×20 magnification. Pulmonary VEGF mRNA was measured by qRT-PCR (**F**). Western blot analyses showed an important decrease of eNOS (**G**) and CD34 (**H**) expression after KYP-2047 treatments compared to BLM group. Data are representative of at least three independent experiments. One way ANOVA test **p < 0.01 vs Sham; ***p < 0.001 vs Sham; ^#^p < 0.05 vs BLM; ^##^p < 0.01 vs BLM; ^###^p < 0.001 vs BLM

Angiogenesis play an important role in the development of pulmonary fibrosis [40], therefore, to emphasize the in vivo modulatory action of KYP-2047 on angiogenesis in lung, an immunohistochemistry analysis of VEGF was performed [13]. The data showed an increase of VEGF positive staining in the middle lobe of the right lung sections of BLM group (Fig. 11B, see VEGF positive score Fig. 11E) compared to sham group (Fig. 11A, see VEGF positive score Fig. 11E). KYP2047 treatments significantly reduced the VEGF positive staining (Fig. 11C and D, see VEGF positive score Fig. 11E). Moreover, as expected the expression of VEGF mRNA was significantly upregulated in BLM-treated mice (Fig. 11F). VEGF stimulates eNOS expression in endothelial cells because it requires nitric oxide to induce angiogenesis [41]. To better understand the ability of KYP-2047 treatment to modulate angiogenesis through the VEGF/eNOS pathway, we evaluated eNOS expression by Western blot analysis. As we expected, in the BLM-induced group we observed a significant increase of eNOS expression compared to the sham group. Interestingly, treatment with KYP-2047 significantly reduced eNOS expression compared to BLM mice (Fig. 11G). We also evaluated CD34 expression as a marker of neoangiogenesis and our results showed a significant increase of CD34 expression in BLM-induced group compared to the sham group. Again, treatment with KYP-2047 significantly reduced CD34 expression, confirming the ability of KYP-2047 to modulate angiogenesis also in a fibrotic condition (Fig. 11H).

#### KYP-2047 modulated JAK2/STAT3 pathway

**Fig. 12:**
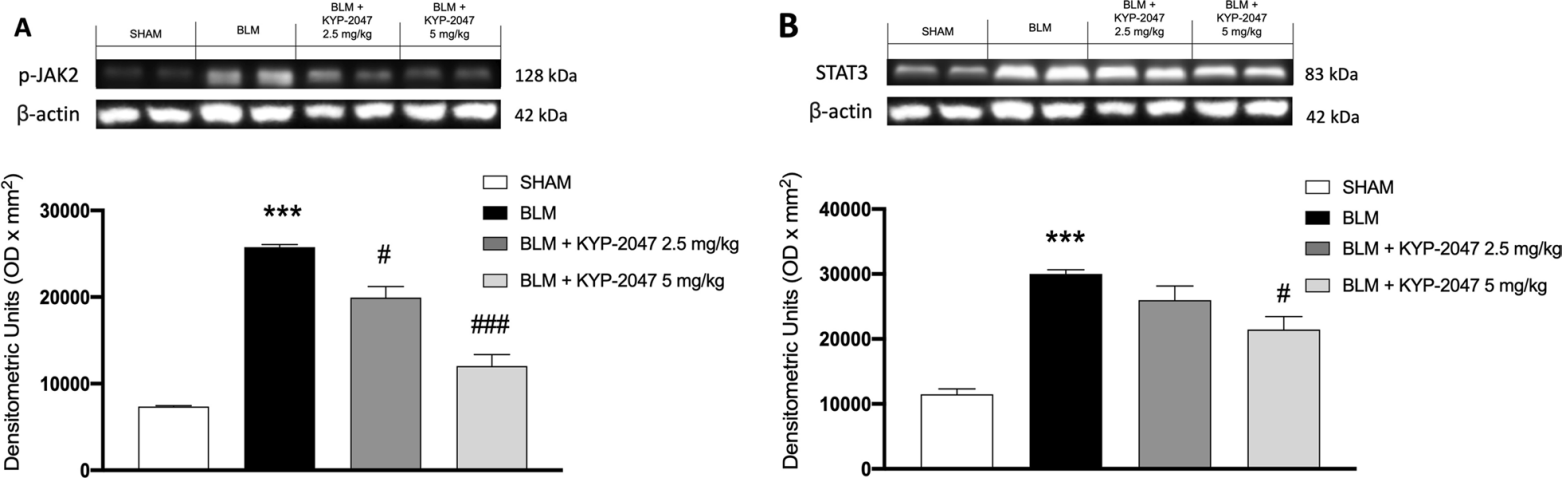
Treatment with KYP-2047 modulated JAK2/STAT3 pathway. Western blot analyses showed a decrease of p-JAK2 (**A**) and STAT3 (**B**) expression after KYP-2047 treatments compared to BLM group. Data are representative of at least three independent experiments. One way ANOVA test ***p < 0.001 vs Sham; ^#^p < 0.05 vs BLM; ^###^p < 0.001 vs BLM

We also investigated one of the main inflammatory pathways induced by pulmonary fibrosis such as the JAK2/STAT3 pathway, to evaluate a possible mechanism of action underlying the beneficial effects of KYP-2047 on pulmonary fibrosis. Western blot analysis revealed markedly increased expression of p-JAK2 and STAT3 in BLM mice compared with sham mice (Fig. 12A and B). Lung tissues from KYP-2047-treated mice showed reduced expression of p-JAK2 at both doses of 2.5 mg/g and 5 mg/kg, compared with BLM mice (Fig. 12A). A significant reduction of STAT3 was observed after treatment with KYP-2047 at the highest dose of 5 mg/kg compared to BLM mice (Fig. 12B).

#### Effects of KYP-2047 on lung inflammation induced by BLM

**Fig. 13:**
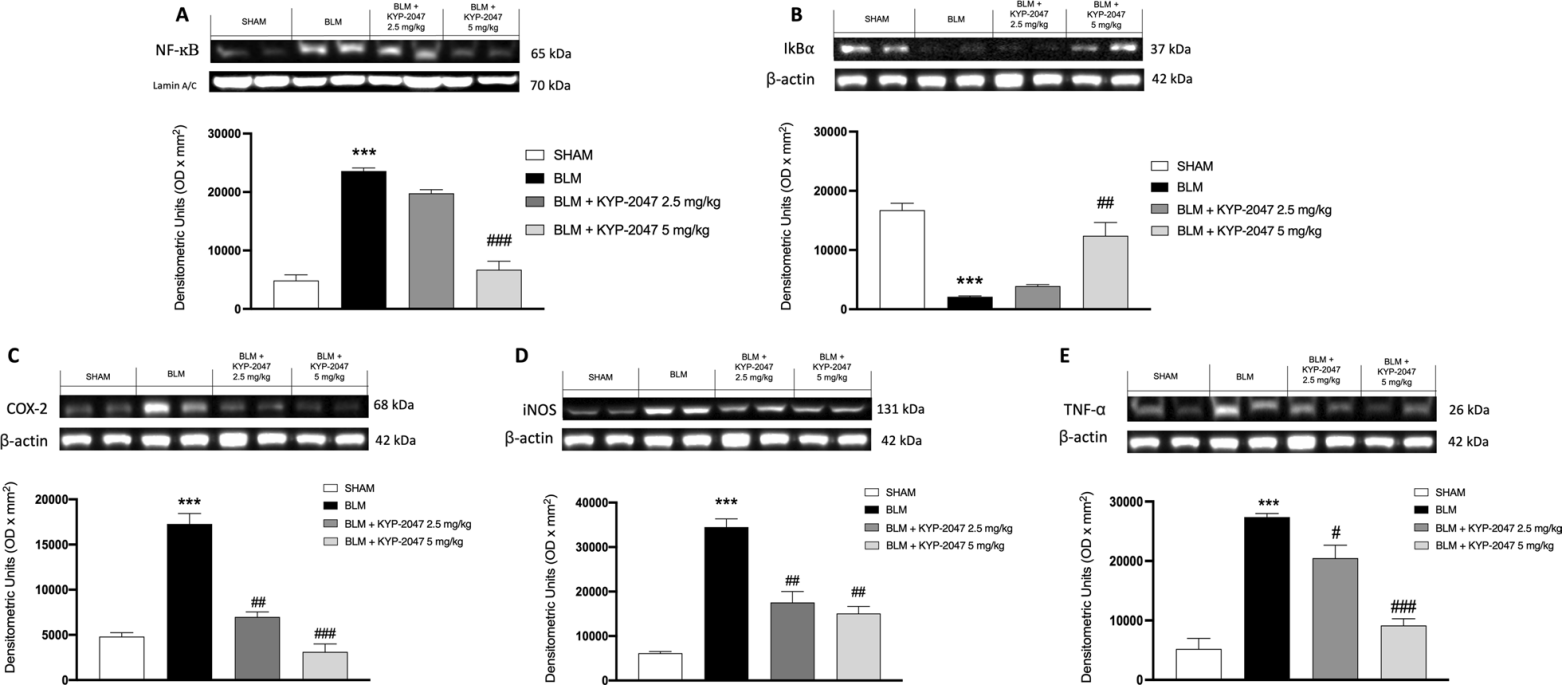
Anti-inflammatory effect of KYP-2047. Western blot analysis showed an important modulation of IκBα/NF-κB pathway (respectively **B** and **A**). Additionally, treatments with KYP-2047 reduced COX-2 (**C**), iNOS (**D**) and TNF-α (**E**) expression compared to the BLM group. Data are representative of at least three independent experiments. One way ANOVA test ***p < 0.001 vs Sham; ^#^p < 0.05 vs BLM; ^##^p < 0.01 vs BLM; ^###^p < 0.001 vs BLM

NF-κB appears to have a pro-inflammatory function in the lung resulting in neutrophilic infiltration and pulmonary edema [13]; moreover, it has been shown that PREP regulate the NF-κB pathway [16]. Therefore, in this study, we confirmed in vivo the modulation of NF-κB pathway by Western Blot analysis. BLM-induced group showed a significant increase of nuclear NF-κB translocation in lung samples compared to control group, while KYP-2047 treatments reduced its expression (Fig. 13A). On the contrary, the IκBα expression was reduced in lung samples of animals subjected to BLM compared to the sham group. KYP-2047 treatment, at both doses of 2.5 and 5 mg/kg, was able to prevent IκBα cytosolic degradation (Fig. 13B). Then, we evaluated the expression of COX-2, iNOS and TNF-α on lung samples by Western Blot analysis. BLM group showed an increased expression of COX-2, iNOS and TNF-α in lung samples compared to sham mice. Whereas KYP-2047 treatment, at both doses of 2.5 and 5 mg/kg, was able to reduce the expression of these inflammatory markers in a significant way (respectively, Fig. 13C–E).

## Discussion

Pulmonary fibrosis is characterized by complex and dynamic interactions between regenerating/reparative epithelial cells and activated fibroblasts [42]. The progression of damage to chronic fibrosis is associated with the epithelial-mesenchymal transition (EMT) of fibroblasts to myofibroblasts, responsible for extracellular matrix synthesis and tissue remodeling. Recent findings indicate that inflammation and angiogenesis alterations are significantly involved in the pathophysiology of IPF [43]. Hypoxic inflammatory tissue, through the upregulation of factors such as VEGF and chemokines, promotes angiogenesis and recruits inflammatory cells [44].

Recent findings have indicated the involvement of the peripheral enzyme PREP in angiogenesis and inflammation. Studies in patients with chronic obstructive pulmonary disease and cystic fibrosis showed that PREP contributed to airway inflammation by generating the neutrophil chemoattractant PGP from lung collagen. These studies were further validated with data obtained in a mouse model of pulmonary emphysema where a significant increase in PREP activity was observed in lung homogenates subject to injury [45–47]. Furthermore, the role of PREP in airway inflammation has been studied in our laboratory in which the treatment with PREP inhibitor, KYP-2047, showed a protective role in acute lung injury induced by intestinal ischemia–reperfusion [13].

Although the exact mechanism is still under investigation, recent studies also demonstrated the ability of PREP to induce angiogenesis, trough the release of pro-angiogenic molecules, such as VEGF [17, 48]. Therefore, based on these evidences, in the present study we investigated the potential properties of PREP inhibition, trough KYP-2047. Lung epithelial cells, especially type II alveolar epithelial cells, are frequent targets of injury and implicated in repairs during progressive fibrotic disorders. Our in vitro model allowed to understand whether PREP could represent a hypothetical target for the treatment of lung diseases. In response to inflammatory stimuli like LPS, the treatment with KYP-2047 increased IκBα expression and significantly reduced NF-kB expression. Inflammation driven by the activation of the IκBα /NF-κB pathway is not only involved in the early stages of IPF, but also plays a key role during the self-repair processes that cause fibrosis. Indeed, alveolar cells that activate NF-κB inflammatory signaling pathways subsequently release a number of proinflammatory factors such as TNF-α and IL-18 that can directly or indirectly promote tissue fibrosis [49]. We found that KYP-2047 was able to reduce the expression of the pro-inflammatory cytokines TNF-α and IL-18 previously overexpressed due to LPS stimulation. Moreover, PREP inhibition reduced the pro-inflammatory enzymes COX-2 and iNOS. As demonstrated by other in vitro studies, treatment with LPS in alveolar epithelial cells generates the free radical NO [50–52]. Moreover, the ability of KYP-2047 in reducing nitrosative stress was showed in vitro by the significant decreasing of NO levels following PREP inhibitor treatment. These in vitro results highlighted the anti-inflammatory effect of KYP-2047 by modulating NF-κB pathway. For these reasons, subsequently, to assess the efficacy of KYP-2047 in a more complex system, we developed an in vivo model of pulmonary fibrosis. Our study demonstrated that treatment with KYP-2047 restored the histological alterations reducing severe lung injury characterized by inflammatory cell infiltration and substantial alveolar edema. One of the characteristics of pulmonary fibrosis is a significant accumulation of fibroblast that synthesize and deposit collagen [13]; our results clearly demonstrated the ability of KYP-2047 to reduce the distribution and content of collagen in the lung, evidencing the protective effect of PREP inhibition in fibrosis-related lung damage. The abnormal repair process responsible for fibrotic damage is driven by hyperactivation of TGF-β, a potent profibrotic cytokine, which promotes EMT of fibroblasts to myofibroblasts that recruit others inflammatory cells [53, 54]; our study demonstrated the ability of KYP-2047 to reduce the fibrotic damage modulating the expression of fibrotic markers such as E-cadherin and N-cadherin. Recent studies have shown a significant expression of TGF-β1 in patients with pulmonary fibrosis [55]; in this regard, our results showed that treatment with KYP-2047 significantly reduced TGF-β1 protein levels in the lung lesion. Furthermore, overexpression of α-SMA actively produces extracellular matrix (ECM) resulting in collagen accumulation [56]. In agreement with this evidence, our results showed an overexpression of α-SMA following induction with bleomycin while treatment with KYP-2047 significantly reduced its expression. Although the etiology remains unknown, aberrant angiogenesis and inflammation play an important role in the development of pulmonary fibrosis [7]. The turnover of the ECM and the consequent destruction of the normal alveolar architecture interrupts the gaseous exchange, establishing a hypoxic microenvironmental condition. In response to the hypoxic condition, cells increase the expression of crucial promoters of angiogenesis such as VEGF and CD34 which promote fibroproliferation and ECM deposition contributing to the progression of IPF [52, 57, 58]. This demonstrates that the pathological angiogenesis involved in IPF could represent a potential therapeutic target in this pathology [59]. In this study we confirmed the involvement of VEGF, eNOS and CD34 in IPF with their overexpression following BLM induction, while treatments with KYP-2047 significantly reduced their expression. Recent studies have shown that STAT3 is activated in fibrotic lung tissue [60] and that it is involved in angiogenesis and inflammatory processes. STAT3 phosphorylation has been shown in fibrotic lung tissue of IPF patients and is involved in lung epithelial cell damage; therefore, could be an interesting therapeutic target in IPF [61]. Indeed, to learn more about the antifibrotic mechanism of PREP inhibition, we investigated JAK2/STAT3 pathway, highly involved in IPF pathogenies [61]. JAK kinases are receptor-associated tyrosine kinases that play important roles in cytokine and growth factor signaling. Binding of JAK2 kinases to pro-inflammatory cytokines induces autophosphorylation and subsequent activation. JAK2, once activated, recruits and phosphorylates STAT3 which dimerizes and translocates into the nucleus where it activates the transcription of several genes involved in the fibrotic response like IL-6 family of cytokines, which signal through STAT3, may also contribute to lung fibrosis [62]. Moreover, JAK2/STAT3 is indicate as a downstream factor in fibrosis, to generate ECM-producing cells by induction of differentiation of organ resident cells via canonical and non-canonical pathways. Several lines of evidence point to the fundamental role of STAT3 in fibroblast plasticity during fibrosis and the pharmacological inhibition of JAK2/STAT3 by KYP-2047 decreased fibrosis in these models [60, 61, 63]. Our results provide evidence that intraperitoneal administration of KYP-2047 was able to reduce the phosphorylation of both JAK2 and STAT3. The modulation of the JAK2/STAT3 pathway could speculate a possible KYP-2047 mechanism of action to exert a protective role in IPF. In addition, considering the role of NF-κB in pulmonary fibrosis [64–66], we investigate the ability of KYP-2047 in modulating IκBα/NF-κB pathway also in BLM model, confirming the results obtained in in vitro. Data from the literature imply that NF-κB drives pulmonary fibrosis as an upstream regulator. In particular, during the initial phases of the fibrotic process, NF-κB increases the expression of genes coding for the synthesis of many inflammatory cytokines, such as TNFα, IL6, IL1β and TGFβ, essential for the development of pulmonary fibrosis. Furthermore, once NF-κB activates the TGF-β/Smad signaling pathway, subsequent activation of the EMT process occurs [67–70]. Our in vivo results confirmed the ability of KYP-2047 treatment to modulate the IκBα/NF-κB pathway and also to reduce the expression of related pro-inflammatory enzymes and cytokines.

## Conclusion

In conclusion, this study demonstrated the involvement of PREP in the pathogenesis of IPF and that PREP- inhibition, mediated by KYP-2047 treatment, has a protective role in lung injury induced by BLM, suggesting PREP as a potential target therapy for IPF. Although the molecular mechanisms underlying the action of KYP-2047 are still to be investigated, these results suggested the modulation of JAK2/STAT3 and NF-κB pathways. For these reason, considering these data as preliminary results, future experiments will be conducted to validate the activity of KYP-2047 on the JAK2/STAT3 pathway using JAK2/STAT3 inhibitors both in vitro and in vivo as well as the use of KO mice.

## Data Availability

The data that support the findings of this study are available from the corresponding author upon reasonable request.

## Abbreviations

IPF: Idiopathic pulmonary fibrosis
PREP: Prolyl oligoendopeptidase
BLM: Bleomycin
LPS: Lipopolysaccharide
KYP-2047: 4-Phenylbutanoyl-l-prolyl-2(S)-cyanopyrolidine
iNOS: Inducible nitric oxide synthase
COX-2: Cyclooxygenase-2
IκB-α: Kappa B-alpha
NF-κB: Nuclear factor kappa-light-chain-enhancer of activated B cells
IL-18: Interleukin-18
TGF-β: Transforming growth factor β
VEGF: Vascular endothelial growth factor
JAK2: Janus kinase 2
STAT3: Signal transducer and activator of transcription 3
α-SMA: α-Smooth muscle actin
eNOS: Endothelial nitric oxide synthase
CD34: Cluster of differentiation
NO: Nitric oxide
